# Mental Health Training Programs for Non-Health Professionals and Volunteers Working with Asylum Seekers and Refugees: A Scoping Review Protocol

**DOI:** 10.3390/nursrep12010010

**Published:** 2022-02-09

**Authors:** Luísa Teixeira-Santos, Filipa Ventura, Luísa Teixeira, Daniela Cardoso, Wilson Abreu

**Affiliations:** 1 Center for Health Technology and Services Research, 4200-440 Porto, Portugal; 2 Health Sciences Research Unit: Nursing, Nursing School of Coimbra, 3046-851 Coimbra, Portugal; filipaventura@esenfc.pt (F.V.); ltrfilipe@esenfc.pt (L.T.); dcardoso@esenfc.pt (D.C.); 3 Nursing School of Porto, 4200-072 Porto, Portugal; wjabreu@esenf.pt

**Keywords:** asylum seekers, education, mental health, refugee, training program

## Abstract

The number of people forcibly displaced worldwide is increasing. It is an imperative challenge to provide mental health training for non-health professionals and volunteers who work with those vulnerable populations. The objective is to identify mental health training programs for non-health professionals and volunteers, without mental health training, to work with asylum seekers or refugees in any context. Literature about mental health training programs for adults, non-health professionals and volunteers without mental health training, working, or who have worked, or will work, with asylum seekers or refugees will be considered. Literature published in English, Swedish, Portuguese, Spanish, and French will be considered. Literature reporting training programs exclusively for professionals or volunteers in the health or mental health domains will be excluded. The following electronic databases will be searched: MEDLINE, CINAHL, PsycINFO, Psychology & Behavioral Sciences Collection, SCOPUS, ERIC, RCAAP, and OPEN GREY. Other sites to be searched: ClinicalTrials, UNHCR, IOM, WHO, Save the Children, IMISCOE, and IFRC. The screening process will entail two steps carried out by two independent reviewers: firstly, screening by title and abstract, and secondarily, by full text. Data will describe the literature according to the review research questions that were defined following the PPC mnemonic, and presented graphically with a narrative.

## 1. Introduction

By the end of 2020, as a result of conflicts and violence, more than 82.4 million people were forcibly displaced worldwide. Of those, more than 26.4 million have refugee status, and 4.1 million seek asylum [1]. Most refugees came from just five countries: 6.8 million from the Syrian Arab Republic; 4.9 million from Venezuela; 2.8 million from Afghanistan; 2.8 million from South Sudan; and 1.1 million from Myanmar. Turkey is the host country that receives the largest number of refugees worldwide, with a registration of 4 million people. Colombia follows with a register of 1.7 million refugees, Germany registers 1.5 million refugees, and Pakistan and Uganda register 1.4 million refugees each [1,2]. The forcibly migratory fluxes are a concern to the European Union. In recent years, the number of people seeking protection in Europe has grown considerably. From 2014 until December 2020, European countries such as Italy, Cyprus, Malta, Greece, and Spain received 2,176,820 million sea and land arrivals [3]. While COVID-19 has temporarily led to a reduction in the number of asylum seekers in the last months, the underlying factors related to global conflicts remain unaddressed [1]. Therefore, it is important to invest in the improvement of host countries’ reception conditions.

Oftentimes, forcibly displaced people must abruptly leave all belongings except the extremely necessary. They do not only lose material resources such as housing, education, and access to health and security, but also identity references, social relationships, and sociocultural supports [4,5]. Forced migration requires multiple adaptations in short periods of time, and people become more vulnerable to mental health problems. The way people are received, protected, and assisted in host countries may aggravate pre-existing problems [6]. Several studies were conducted to understand the impact of those kinds of situations on asylum seekers’ and refugees’ mental health. A recent systematic review and meta-analysis on 26 studies that provides results about 5143 adult refugees and asylum seekers, from all over the world, concludes that refugees and asylum seekers have high and persistent rates of post-traumatic disorder, depression, anxiety, and psychosis, and highlights the need for ongoing, long-term mental health care [7]. These rates have been linked to traumatic events such as violence, separation, sexual abuse, trafficking, harassment, and lack of basic needs [8,9,10]. Moreover, the mental health of refugees seems to be distinct from the experiences of other traumatized populations. such as veterans or sexual assault victims, due to their specific traumatic experiences and the stress in the acculturation process after resettlement related to new cultures, practices, settings, and lack of familiar support systems [11]. As a consequence of some countries’ political arrangements, the asylum seekers are getting stopped at the borders. If arriving in a host country, they must wait a long period to obtain refugee status, oftentimes in untenable conditions, which leads to an increased vulnerability in mental health [4,12]. To wait for refugee status, asylum seekers are escorted to common facilities, reception centers, community shelters, refugee camps, or camps with surveillance. The conditions in such locations are highly variable, but most of them are inhumane [13].

The United Nations High Commissioner for Refugees (UNHCR) maintains strategic partnerships with more than 900 partners, most of whom are Non-Governmental Organizations (NGOs) [14], with the purpose to safeguard the rights and well-being of refugees. Volunteers from civil society and from local or international NGOs are the main group working with asylum seekers and refugees in the reception locations guaranteeing the chain of humanitarian assistance, solidarity, and inclusion in society [15]. Even with a high capacity for self-help and resilience, staff and volunteers aiding asylum seekers and refugees on the move are repeatedly exposed to personal tragedies. They may also live and work under physically and psychologically demanding and unpleasant working conditions [6,16]. Volunteers might experience moral anguish over the choices they have to make, increasing adverse consequences such as anxiety and depressive feelings, over-involvement with beneficiaries, callousness, apathy, self-destructive behavior, and interpersonal conflict [6]. Not only for themselves, but also to provide better and adequate care for the asylum seekers and refugees who wait for their refugee legal status for long periods in reception centers, shelters, or refugee camps, it is important to invest in some mental health basic competencies that empower them to make earlier and correct decisions. Improving the skills and knowledge of volunteers, who are non-health professionals, in mental health management will have a very positive impact in terms of prevention, early detection and appropriate referral, reducing stigma and discrimination, and improving their rights [17,18,19]. For the UNHCR, mental health and wellbeing psychosocial support primary activities with refugees and asylum seekers can be provided by people who are not specialized in the field, but who have been trained and supervised [19].

The research on the volunteers, who are often some of the first people to develop close contact with asylum seekers and refugees, is increasing slowly [18], and lacks the systematic overview to understand what kind of mental health competencies training the volunteers have that allows them to work with asylum seekers and refugees. Mental health competence is understood as the ability to participate effectively in efforts to promote prevention, care, treatment, and advocacy for mental health. As volunteers and non-health professionals working with asylum seekers, they should have knowledge and skills to recognize people’s suffering based on cultural competencies. Mental health competence also requires empowerment skill to help these vulnerable populations daily and help them to seek specialized mental health professionals. 

A review is needed to systematize the mental health training programs that are carried out, in which specialist mental health nurses can contribute and improve. Thus, this scoping literature review aims to identify and describe the mental health training programs for non-health professionals and volunteers who work, have worked, or will work with asylum seekers or refugees in any context. The review seeks to examine whether there are different types of mental health training programs and courses for civil society, who do not have a health domain background, but need to develop those skills to deal with asylum seekers and refugees. The review will also be fundamental to understanding the settings in which these training programs take place, who conducts these mental health training programs, and the kind of educational domains and strategies used.

This aim was established following the Participants, Concept, Context (PCC) mnemonic as recommended by Joanna Briggs Institute for scoping reviews [20]. A preliminary search of PROSPERO, MEDLINE, the JBI Evidence Synthesis, and OFS was conducted, and no current or underway scoping reviews on the topic were identified.

## 2. Materials and Methods

### 2.1. Review Questions

What are the mental health training programs that have been used in the preparation of non-health professionals and volunteers, who do not have mental health training, to work with asylum seekers and refugees in the most varied contexts?

This review will seek to describe the training programs by answering the following sub-questions:

(a) Who are the trainers conducting the training programs in mental health competencies for non-health professionals and volunteers working with asylum seekers and refugees?
(b) At which settings are the mental health training programs for non-health professionals and volunteers working with asylum seekers and refugees occurring?
(c) What are the educational domains composing the training programs in mental health competencies available for non-health professionals and volunteers working with asylum seekers and refugees?
(d) What pedagogical strategies are being used for the training of non-health professionals and volunteers working with asylum seekers and refugees in mental health competencies?

### 2.2. Inclusion Criteria

#### 2.2.1. Participants

This review will consider studies that include literature reporting on training in mental health competencies of adults aged ≥18 years, non-health professionals and volunteers, who do not have mental health training, and who work, are working, or will work with asylum seekers or/and refugees. It will include studies with participants independent of their educational level, who may have received or are receiving training in mental health competencies. The studies retrieving training programs exclusively for health personnel or mental health professionals, such as psychologists, will be excluded. Although the studies cannot be specifically for people with a health degree or mental health background, studies about mental health training programs for volunteers of civil society which have in their sample health professionals or people with mental health backgrounds (e.g., programs for volunteers independent of their professional background) will be included. 

#### 2.2.2. Concept

This review will consider literature that reports training programs aiming to improve mental health competencies of non-health professionals and volunteers. For the purpose of the current review, a mental health training program is considered to be any course or program carried out by trainers (e.g., health or social sciences educators, NGO professionals, or NGO volunteers), irrespective of the duration, with the aim of developing or improving the competencies in the mental health domain. The educational strategy might include face-to-face or online programs, with or without practical training to develop or improve mental health competencies. This review will consider mental health competencies to be defined as the knowledge, skills, abilities, and personal attributes of non-health professionals and volunteers to effectively identify people experiencing mental health suffering and in need of professional support, and provide mental wellbeing support as non-health professionals or volunteers. The analysis of the identified training programs in mental health will constitute the basis to map and understand their characteristics in relation to the educational domains, strategies, and settings, as well as the trainers and trainees guiding their structure.

#### 2.2.3. Context

This review will consider literature that focus on the mental health training performed irrespectively of the settings, including but not limited to NGOs, host countries, governmental institutions who receive and support asylum seekers or refugees, community contexts, universities, polytechnics, shelters, detention centers, and refugee camps. Literature will be included irrespective of geographic location without a specific racial- or gender-based criteria.

#### 2.2.4. Types of Sources

This scoping review will consider primary studies, quantitative, qualitative, mixed- and multi-method study designs, and reviews for inclusion in several databases described in the search strategy section. In addition, several relevant websites will be searched to identify information that will not be available in scientific databases, namely websites of reputable non-governmental organizations and websites that provide studies in progress. This information is more fully described in Section 2.3.1. Conference abstracts and text opinion papers will also be considered for inclusion in the proposed scoping review. 

### 2.3. Methods

The proposed scoping review will be conducted in accordance with the JBI methodology, chapter 11, for scoping reviews [12], which encompasses the work of Arksey and O’Malley [14] with refinements by Levac, Colquhoun and O’Brien [15]. The development of the protocol for the current review complies with the Extension for Scoping Reviews of the Preferred Reporting Items for Systematic Reviews and Meta-Analyses (PRISMA-ScR) [16].

#### 2.3.1. Search Strategy

The search strategy will aim to locate both published and unpublished primary studies. An initial limited search of MEDLINE (PubMed) and CINAHL (EBSCO) was undertaken to identify articles on the topic. Furthermore, a search in the WHO website was also made. The text keywords contained in the titles and abstracts of relevant articles, and the index terms used to describe the articles, were used to develop a full search strategy for MEDLINE (EBSCO), CINAHL (EBSCO), PsycINFO, Psychology & Behavioral Sciences Collection, SCOPOUS, ERIC, RCAAP, and OPEN GREY (see Table A1 Appendix A). All articles available up to the day of the database search will be included. 

The search strategy, including all identified keywords and index terms, will be adapted for each included information source. The reference lists of articles included in the review will be screened for additional papers. The search will also include the websites of ClinicalTrials, United Nations High Commissioner for Refugees (UNHCR), International Organization for Migration (IOM), World Health Organization (WHO), Save the Children, International Migration, Integration and Social Cohesion in Europe (IMISCOE), and International Federation of Red Cross and Red Crescent Societies (IFRC). The NGO websites listed as sources of information were provided by a WHO Guidance Note that identify them as NGOs with developed work on protecting and supporting the mental health and psychosocial wellbeing of refugees, asylum seekers, and migrants [6].

Articles published in English, Swedish, Portuguese, Spanish, and French, without a time range, will be included. 

#### 2.3.2. Study/Source of Evidence Selection

Following the search strategy presented above, all identified records will be collated and uploaded into EndNote™ X8 (Clarivate Analytics, Philadelphia, PA, USA) and duplicates removed. Following a pilot test, titles and abstracts will then be screened by two independent reviewers for assessment against the inclusion criteria for the review. Potentially relevant papers will be retrieved in full and their citation details imported into the JBI System for the Unified Management, Assessment and Review of Information (JBI SUMARI; JBI, Adelaide, Australia) [21]. The full text of selected citations will be assessed in detail against the inclusion criteria by two independent reviewers. Reasons for exclusion of full-text papers that do not meet the inclusion criteria will be recorded and reported in the scoping review. Any disagreements that arise between the reviewers at each stage of the selection process will be resolved through discussion or with a third reviewer. The results of the search will be reported in full in the final scoping review and presented in a PRISMA-ScR flow diagram [22].

#### 2.3.3. Data Extraction

Data will be extracted from papers included in the scoping review by two independent reviewers using a data extraction tool developed by the reviewers. The data extracted will include specific details about the mental health training programs available for non-health professionals and volunteers, without mental health training, who have worked, work, or will work with asylum seekers, irrespective of settings where the training might occur, and other relevant findings to the review question. The data will be exported to Microsoft Excel^®^ (Redmond, WA, USA). A draft extraction tool is provided (see Table A2 in Appendix A). The draft data extraction tool will be modified and revised as necessary during the process of extracting data from each included paper. Modifications will be detailed in the full scoping review. Any disagreements that arise between the reviewers will be resolved through discussion or with a third reviewer. Authors of papers will be contacted to request missing or additional data, where required.

#### 2.3.4. Data Analysis and Presentation

Results will be reported graphically with tables when possible. Tables will be developed and refined throughout the data extraction to reflect the purpose and objective of the review. The results will be classified under the following categories: study identification (ID); reasons for inclusion or exclusion; characteristics of study population/paper, regarding the participants, settings, educational mental health domains, and the strategies that are being used for the training; and a category about research methods used in the study/paper.

A narrative will accompany the result tables and will describe the characteristics of the body of literature related to mental health training programs available for the training of non-health professionals and volunteers, without mental health training, in any context. Data synthesis and analysis will be conducted using a thematic analysis. 

## 3. Results

Most of the existing mental health training programs are developed for health professionals, often targeting specific mental health diagnoses, or do not contemplate the mental health of people under 18 years. With the proposed scoping review, we will map and synthesize the evidence regarding the mental health training programs for non-health professionals and volunteers working with young asylum seekers. With this work, we will also identify the mental health trainers conducting these kinds of programs for the population of interest and point out the setting used for the training. In addition, we expect to identify which educational domains are included in the programs and which pedagogical strategies are used. 

## 4. Conclusions

With the overwhelming growth of forcibly displaced people worldwide, it is necessary to understand what mental health training is received by non-health professionals and volunteers who work with people living the most traumatizing moments of their lives. To map these mental health training programs will not only help us to identify the mental health training programs available in databases, but also in the sites of the most relevant NGOs working with displaced people. It is crucial to synthesize this evidence in a scoping review, not only to understand if the mental health training programs for non-health professionals and volunteers exist, but also to understand how and by whom they are conducted, and what mental health competencies are learned. 

## Data Availability

No new data were created or analyzed in this study. Data sharing is not applicable to this article.

## Appendix A

**Table A1:** Search Strategy for MEDLINE (EBSCO) Search conduct on 16 December 2021.

Search	Query	Records Retrieved
#1	MH “Mental Health” OR TI Mental OR AB mental OR TI (well-being or wellbeing or “well being”) OR AB (well-being or wellbeing or “well being”)	481,858
#2	MH education OR TI (training * OR course * OR “educational model *” OR program * OR approach * OR procedure * OR method * OR strateg *) OR AB (training * OR course * OR “educational model *” OR program * OR approach * OR procedure * OR method * OR strateg *)	7,846,056
#3	MH Refugees OR MH “Refugee Camps” OR MH “United Nations” OR TI (refugee * OR “asylum seeker *” OR “forced migrant *”) OR AB (refugee * OR “asylum seeker *” OR “forced migrant *”)	23,658
#6	#1 AND #2 AND #3	1553
Limited to English, Swedish, French, Portuguese, Spanish		1511

# = Research line, * = Truncation.

**Table A2:** Data Extraction instrument.

Study ID	Study Number	
	2. Authors	
	3. Year	
	4. Article Title	
	5. Journal	
	6. Issue no.	
	7. Vol.	
Reason for inclusion or exclusion	8. Did the study or source of information present a mental health training program?	1: Yes 2: No—exclude
	9. Did the study or source of information involve training programs for non-health professionals and volunteers, without mental health training, who have worked, who work, or will work with asylum seekers or refugees?	1: Yes 2: No—exclude
	10. Did the literature include the training of mental health competencies provide by professionals with training?	1: Yes 2: No—exclude
	11. Are there other reasons for exclusion?	1: Yes 2: No
	11.1. Specify other reason for exclusion	Specify in own words reason for exclusion
	12. Inclusion of paper?	1: Yes 2: No
Characteristics of study population/paper	13. Which non-health professionals and volunteers are target in the study?	1. Non-specified 2. Other, specify
	13.1. Sample size	Specify the number
	14. Who are the trainers conducting the mental health training programs?	1. Medical Staff 2. Nurses 3. Psychologists 4. Social Workers 5. Health educators 6. Midwifes; 7. Sociologists 8. Anthropologists 9. Multidisciplinary team 10. NGO’s professionals 10.1. Describe which NGO 11. Other, specify
	15. Setting of the study or settings where supposed to teach the mental health training program	1. Recreational/cultural groups that supports asylum seekers and refugees locally 2. Refugee camps 3. NGOs 4. Universities and Polytechnics 5. Detention Centers for migrants 6. Refugee camps 7. Prison or custodial settings 8. Other, please specify
	16. Name of the program	Specify the name
	17. The domains included in the mental health training program	1. Transcultural Mental challenges 2. Prevalence Mental Health Disorders 3. Psychological Support 4. Social Support 5. Values and Beliefs in mental health assistance 6. Communication 7. conflict resolution 8. Ethical and moral approach of asylum seekers and refugees 9. Exercises to practice mental health competencies 10. Other, please specify
	18. The strategies used in the training program	1. Mandatory Participation 1.1. Online 1.2. Face-to-Face 2.Expository Method 3. Role Play 4. Practical interaction with asylum seekers and refugees in the fieldwork 5. Other, please specify
	19. There are any models or theories or principles or specific guidelines used to conceptualize the training	1. Yes, Please describe. 2. Not described
Research methods used in the study/paper or description of other sources. Studies will be categorized according to the methodology or methodologies employed, and whether or not the data collected is numeric.	20. Is this training programs provided by NGOs or International organizations considered relevant for the topic?	1: Yes 2: No
	21. Is the study experimental?	1: Yes 2: No
	21.1. If experimental—what type?	1. Classic experiment/RCT 2. Experiment without randomization 3. Experiment without control group 4. Not experimental
	21.2. What instruments are used to measure outcomes?	Please identify the instruments (i.e., Scales, Questionnaires)
	22. Is the study observational?	1: Yes 2: No
	22.1. If observational—what type?	1. Correlational (retrospective) 2. Correlational (prospective) 3. Cross sectional 4. Case control 5. Other Descriptive 6. Not observational
	23. Is it a qualitative study?	1: Yes 2: No
	23.1. How is data collection performed?	Please describe them (e.g., Focus groups, interviews)
	24. Is the study is a multi-method study?	1: Yes 2: No
Summary the study	25. Make a short summary of the article.	Describe them.
Comments to review from reviewers	Describe them.	
